# A new diagnostic approach in Alzheimer's disease: The critical flicker fusion threshold

**DOI:** 10.1590/1980-5764-DN-2021-0054

**Published:** 2022

**Authors:** Azar Abiyev, Funda Datlı Yakaryılmaz, Zeynel Abidin Öztürk

**Affiliations:** 1 Gazi University, Faculty of Medicine, Department of Gastroenterology, Ankara, Turkey.; 2 Malatya Training and Research Hospital, Department of Geriatrics, Malatya, Turkey.; 3 Gaziantep University, Faculty of Medicine, Department of Geriatrics, Gaziantep, Turkey.

**Keywords:** Dementia, Alzheimer DIsease, Critical Flicker Fusion, Demência, Doença de Alzheimer, Fusão de Cintilação Crítica

## Abstract

Alzheimer's disease (AD) is the most common cause of dementia in the elderly. Although AD treatment is still insufficient despite all the recent developments, detection and treatment in the early stage of disease have provided more clinical benefits.

**Objective:**

In this study, we aimed to use the critical flicker fusion (CFF) threshold test to diagnose AD in the early stage.

**Methods:**

In this study, 120 patients (above 65 years of age) and 50 control groups who were admitted to geriatrics outpatient clinic and diagnosed in early- and middle-stage AD were included. The remaining 58 patients and 25 healthy volunteers underwent comprehensive geriatric assessment and CFF testing.

**Results:**

The mean CFF value of AD group was significantly lower than the control group (36.44±7.00 vs. 44.24±3.82, p<0.001, respectively). There was a significant difference in standardized mini-mental state examination (MMSE) score in both groups (18.05±5.25 vs. 25.96±2.85, p<0.001, respectively). There was also a positive correlation between CFF value and MMSE score (p<0.001, r=0.459). Thirty-four patients were in the early-stage AD group and 24 patients were in the middle-stage AD group. There was a significant difference in CFF values between the three groups when we compared the patients in early- and middle-stage AD and control groups (p<0.001). The mean CFF values in patients with early- and middle-stage AD were 37.93±7.33 and 34.97±7.43, respectively. The mean age, gender, education level, and the number of drugs used did not show a statistically significant difference in both groups (p>0.05). The cutoff value for the CFF variable was determined as 39 Hz [p<0.001; area under the curve (AUC)=0.852; sensitivity=70.69% (95% confidence interval [95%CI] 57.3–81.9); specificity=92.00% (95%CI 74.00–99.00)].

**Conclusions:**

There is a significant difference in mean CFF values between AD and healthy groups. CFF testing may play an important role in diagnosing AD in the early stage.

## RESUMO.

A doença de Alzheimer (DA) é a causa mais comum de demência em idosos. Embora o tratamento da DA ainda seja insuficiente mesmo com todos os desenvolvimentos recentes, a detecção precoce e o tratamento no estágio inicial da doença têm demonstrado maior benefício clínico.

**Objetivo:**

Neste estudo, nosso objetivo foi usar o teste *Critical Flicker Fusion Threshold* (CFF) para diagnosticar a DA em estágio inicial.

**Métodos:**

Foram incluídos 120 pacientes e 50 controles em ambulatório de geriatria, com diagnóstico de DA inicial e moderada e acima de 65 anos. Os 58 pacientes restantes e 25 voluntários saudáveis foram submetidos a avaliação geriátrica abrangente e ao CFF.

**Resultados:**

A média de CFF do grupo AD foi significativamente menor do que a do grupo controle (36,44±7,00 *vs*. 44,24±3,82, p<0,001, respectivamente). Houve diferença significativa na pontuação do Miniexame do Estado Mental (MMSE) em ambos os grupos (18,05±5,25 *vs*. 25,96±2,85, p<0,001, respectivamente). Também houve correlação positiva entre o valor CFF e o escore MMSE (p<0,001, r=0,459). Trinta e quatro estavam no estágio inicial e 24 estavam no estágio moderado do grupo DA. Houve diferença significativa nos valores de CFF entre os três grupos quando comparamos os pacientes com DA inicial e moderada e os grupos controle (p<0,001). O valor médio de CFF em pacientes com DA em estágio inicial foi de 37,93±7,33 e de 34,97±7,43 nos pacientes em estágio moderado. As médias de idade, sexo, escolaridade e número de medicamentos usados não apresentaram diferença estatisticamente significativa entre os dois grupos (p>0,05). O valor de corte para a variável CFF foi determinada como 39 Hz (p<0,001; área sob a curva — AUC=0,852; sensibilidade=70,69% [intervalo de confiança de 95%— IC95% 57,3–81,9]; especificidade=92,00% [IC95% 74,00–99,00]).

**Conclusões:**

Há diferença significativa entre os valores médios de CFF do grupo de DA e do grupo saudável. O CFF pode desempenhar um papel importante no diagnóstico de DA no estágio inicial.

## INTRODUCTION

According to the 2015 World Alzheimer's Report, 46.8 million people worldwide were living with dementia, and the total global social cost of dementia was estimated to be US$ 818 billion. Alzheimer's disease (AD) is the most common form of dementia and may account for 60–70% of dementia cases^1^. Initially, AD typically occurs as a progressive memory loss; this accompanies or follows other cognitive dysfunctions such as visual-spatial abnormalities, navigation difficulties, executive problems, and language impairment. These cognitive disorders affect the activities of daily living (ADLs), and most of the behavioral and psychological symptoms of dementia usually occur during the course of the disease.

Pathological evidence for AD suggests that degeneration in cholinergic neuron-rich regions, such as the nucleus basalis of Meynert, frontal cortex, anterior cingulate cortex, and posterior cingulate cortex, is associated with memory loss, agitation, and apathy^2^. Acetylcholine levels have been highly correlated with memory functions, including memory coding, consolidation storage, and retrieval^3^. Pharmacological treatment with acetylcholinesterase inhibitors provides symptomatic benefits in the middle stage of AD, improving the measures of cognition, function, and behavior^4,5^. Starting treatment at an earlier stage aims to preserve cognitive and functional abilities at the highest possible level when impairments are mildest^6^. Therefore, there is a need for a noninvasive, easy-to-use, and inexpensive diagnostic method for detecting early AD.

Neurophysiological techniques, together with minimal invasiveness, are suitable methods for the investigation of patients with AD, as they allow direct measurement of neural activity^7^. Critical flicker fusion threshold (CFFT) is a well-known neurophysiological technique that has been extensively studied in young and elderly healthy volunteers^8^. The neurophysiological basis of flicker perception is complex; however, it is an essential component of visual perception. The CFFT represents the fastest flashing speed of light that the visual system can resolve. Unlike other static visual variables, CFFT appears to be more strongly affected by neural processes at the cortical level^9^. Two previous studies have shown that the CFFT is lower in individuals with AD^8,10^. In this study, we investigated the CFF test for the early detection of AD and then revealed the standardized cutoff values.

## METHODS

### Patients

This cross-sectional study was conducted in patients with AD who were admitted to Gaziantep University Shahinbey Research and Application Hospital of geriatrics outpatient clinic between March 6, 2019 and June 1, 2019, and age, sex, and education level were matched with healthy subjects.

A total of 120 patients were diagnosed with AD according to the diagnostic criteria of the National Institute on Aging and Alzheimer's Association, and 50 healthy individuals were evaluated. Clinical assessments included psychiatric history and mental status examination, physical examination, and comprehensive geriatric assessment tests. In addition, additional information was obtained from the patient's relatives and the caregiver. Laboratory tests and neuroimaging were performed to exclude other causes of dementia. The relative or caregiver received an information form about the study and provided written consent. In the AD group, 44 patients had exclusion criteria, such as visual impairment, 10 patients did not complete the tests, and 8 patients had high standard deviation after the CFF test. In the control group, 20 patients had exclusion criteria and 5 patients did not complete the tests. Consequently, 58 AD patients and 25 healthy subjects were included. AD patients were evaluated in two groups, such as early and moderate stages. Thirty-four patients were in the early stage, while 24 patients were in the moderate stage.

Anthropometric measurements, body composition by bioimpedance analysis, CFF test, and a comprehensive geriatric evaluation in terms of geriatric syndromes, such as sarcopenia, dementia, malnutrition, and falls, were performed on individuals in both groups.

Our study was approved by the decision of Gaziantep University Clinical Research Ethics Committee (dated March 6, 2019 and No. 2019/37).

### Inclusion criteria

Patients >65 years of age and who were in the diagnosis of early- and moderate-stage AD were included.

### Exclusion criteria

Patients having visual and hearing impairment, end-stage cardiovascular and respiratory diseases, chronic liver disease, severe metabolic disorders, and advanced stage of dementia, and using drugs affecting central nervous system were excluded.

### Comprehensive geriatric assessment tests

The detailed history of patient was collected by using several clinical testing modalities, including the geriatric depression scale (GDS), mini-mental state examination (MMSE), Katz index of activities of daily living (ADLs), Lawton–Brody index of the instrumental activities of daily living (IADLs), mini nutritional assessment tool-short form (MNA-SF), and “Tinetti Balance-Gait Evaluation Scale” test (TBGES).

The GDS scores of 5 and above indicate depression^11^. MMSE assesses five different areas in cognitive functions, such as orientation, registration, attention, calculation, recall, and language. MMSE is evaluated out of 30 points. A score of 24–30 for normal, 20–24 for mild, 10–19 for moderate, and 0–9 for severe dementia is observed^12^. According to these scores, we classified the patients with dementia into early and moderate stages.

Katz index of ADLs was used to evaluate the physical disability of elderly individuals. This scale includes bathing, dressing, toileting, transferring, continence, and feeding. Scores can range from 0 to 6, and higher scores indicate higher independence^13^.

Lawton-Brody index was used to evaluate the disability in IADL, and this scale finds out subject performance in the following activities, such as doing laundry, shopping, taking medicine, housekeeping, preparing food, using the telephone, using transportation, and managing money. Higher scores indicate higher independence^14^.

The nutritional status of participants was determined by using MNA-SF. It is a simple and validated screening tool for nutritional risk, and if the score was ≤7, it was accepted as malnutrition^15^.

The risk of falls was evaluated by using the TBGES (Tinetti, 1986). Two subtests of Tinetti are balance and gait. A combined score of the two sections is obtained: a score of >24 results in a low risk of falls, a score from 19 to 23 results in a moderate risk of falls, and, finally, a score of <19 results in a higher risk of falls^16^.

### Anthropometric parameters

Current weight, height, body mass index (BMI), waist circumference (WC), hip circumference (HC), bilateral mid-upper arm (MAC), and calf circumferences (CCs) of all participants were measured using a digital scale with an accuracy of 0.1 kg and a standardized stadiometer with an accuracy of 0.1 cm. Participants removed their socks, shoes, and heavy clothes before the measurements were taken. BMI was defined as person's weight in kilograms divided by the square of person's height in meters. WC was measured at the smallest abdominal girth or in obese individuals in the middle between the lowest rib and the iliac crest. HC was measured horizontally at the point of largest lateral extension at the hips or over the buttocks. CC was measured at the level of the widest circumference of the calf when the participants were standing. MAC was measured between the acromion and the olecranon process at the middle point while elevating and internally rotating the arm. All measurements were conducted by an experienced staff.

### Body composition

A bioelectrical impedance analyzer (BIA) (Tanita SA165 A-0950U-3) was used to assess body composition parameters. BIA was performed after fasting for a minimum of 2 h in an empty bladder. Absolute skeletal muscle mass was estimated using the predictive equation described by Janssen^17^. Skeletal muscle mass index (SMMI) was calculated as absolute skeletal muscle mass (kg)/height squared (m^2^)^18^. The cutoff values for the Turkish population were used for describing low SMMI such as 9.2 and 7.4 kg/m^2^ in men and women, respectively^19^, defined by Bahat et al. Fat-free mass (FFM), referring to all body components except fat, was measured by BIA.

### Muscle strength

Handgrip strength of the dominant hand was measured to determine muscle strength. We used a Jamar hydraulic dynamometer with a validated protocol^20^. The measurement was conducted in sitting position, elbow in 90° flexion, and wrist in neutral position. Participants, who were in sitting position, were squeezed as hard as possible three times (with 30 s rest between each attempt), and the highest was recorded as handgrip strength. As recommended for the Turkish population by Bahat et al., the cutoff thresholds for handgrip strengths, such as 32 and 22 kg for men and women, were used for the diagnosis of sarcopenia^19^.

### Critical Flicker Fusion Threshold

Before the CFF test, the individual was informed how the test was performed. The CFF equipment used is portable and simple to use. The CFF device is connected to the computer and is managed by the computer program. The test subject is given glasses with visual alerts connected to the same device and a button is used to stop the recording. In the middle of the glasses, there is a red light flickering at variable frequency. In the study, psychometric test was performed on patients and healthy individuals in a well-lit, silent room. The test duration was about 10–15 min for each patient or healthy control group. The CFFT was measured by applying binocular foveal stimulation with a red light-emitting diode. Continuous psychophysical limit method was used. The frequency of the light has been reduced down to 60 Hz (descending mode). When the red light seemed to flicker, the individual was informed to push the button. The frequency at which light is perceived as pulsating was determined as the CFFT. This test was performed eight times for each patient, and the mean value was determined.

### Statistical analysis

The Shapiro-Wilk test was used to determine the normal distribution of the parameters of the groups. Descriptive statistics were given as mean and standard deviation for the continuous structure data in the normal distribution groups and median and percentage values for the nonmatching groups. Number and percentage values are given for categorical parameters. Student's *t*-test was used to compare the mean CFF values of participants with and without dementia according to gender, age group, educational level, and the number of drugs used. ANOVA test was used to compare the test results. In addition, Receiver Operating Characteristic (ROC) analysis was performed to determine the CFF cutoff point according to disease status. The relationships between the groups and categorical parameters were analyzed using χ^2^ analysis. The value of p<0.05 was statistically significant. SPSS 11th package program was used for statistical analysis.

## RESULTS

Fifty-eight patients and 25 healthy subjects were included in this study. Out of 58 patients, 26 patients were men and 32 patients were women. The mean age was 70.1±7.6 years. The control group consisted of 13 men and 12 women. The mean age in the control group was 68.5±6.4 years. At the same time, patients with AD were divided into two groups, such as early and moderate stages. Thirty-four patients were in the early stage and 24 patients were in the moderate stage. The marital status, educational level, and habits of the individuals in the AD and control groups are shown in Table 1.

**Table 1:** Sociodemographic characteristics of individuals.

		AD group (%)	Control group (%)	Total
Level of education (with numbers and percentage)	No formal education	23 (79.3)	6 (20.7)	29
	Primary school	25 (71.4)	10 (28.6)	35
	Middle school	1 (25.0)	3 (75.0)	4
	High school	5 (55.6)	4 (44.4)	9
	University	4 (66.7)	2 (33.3)	6
Marital status (with numbers and percentage)	Married	39 (67.2)	19 (32.8)	58
	Others	19 (76.0)	6 (24.0)	25
Smoking (with numbers and percentage)	Yes	16 (64.0)	9 (36.0)	25
	No	42 (72.4)	16 (27.6)	58
Alcohol (with numbers and percentage)	Yes	3 (50.0)	3 (50.0)	6
	No	55 (71.4)	22 (30.1)	77

AD: Alzheimer's disease.

The baseline clinical characteristics and CFF results of both groups are demonstrated in Table 2. The mean CFF value of the AD group was 36.44±7.00 Hz and that of the control group was 44.24±3.82 Hz. There was a significant difference between the mean CFF value of AD and control groups (p<0.001). The mean CFF value of both groups was positively correlated with MMSE and IADL (p<0.001, r=0.459; p<0.05, r=0.231, respectively). There was no significant relationship between the CFF values of both groups with ADL, GDS, MNA-SF, and TBGES.

**Table 2:** Demographic characteristics and anthropometric parameters of study population.

	AD (n=58)	Control (n=25)	p-value
Age	70.14±7.640	68.56±6.417	0.369
BMI (kg/m^2^)	28.49±5.33	28.78±4.93	0.819
CC (cm)	39.18±6.46	43.04±6.59	0.018
HGS (kg)	20.29±11.24	28.35±15.32	0.012
ADL	5.28±1.07	5.72±0.89	0.073
IADL	4.62±2.27	6.68±1.95	<0.001
MMSE	18.06±5.25	25.96±2.85	<0.001
GDS	5.18±3.95	4.25±2.52	0.293
MNA-SF	10.47±2.58	11.52±2.12	0.076
TBGES	21.59±6.25	23.84±5.61	0.125
Mean CFF (Hz)	36.44±7.00	44.24±3.82	<0.001

AD: Alzheimer's disease; BMI: body mass index; CC: calf circumference; ADL: Activities of Daily Living; IADL: Instrumental Activities of Daily Living; MMSE: Mini-Mental State Examination; GDS: Geriatric Depression Scale; MNA-SF: Mini Nutritional Assessment Tool Short Form; TBGES: Tinetti Balance-Gait Evaluation Scale; CFF: Critical Flicker Fusion.

All participants were evaluated in three groups, such as early-stage AD, moderate-stage AD, and control group, and the post hoc analysis was performed. There was a significant difference between the mean CFF values of the three groups (p<0.001). When the three groups were evaluated mutually, there was a significant difference between the mean CFF values of the control group, the early-stage AD group, and the moderate-stage AD group. However, there was no statistically significant difference between the mean CFF values in the early- and middle-stage AD groups (Table 3).

**Table 3:** Mutual evaluation of Critical Flicker Fusion levels of three groups.

		Average difference	Standard deviation	p-value	95%CI	
					Lower limit	Upper limit
Control	Early-stage AD	4.4588*	1.6784	0.026	0.451	8.467
	Moderate-stage AD	7.4138*	1.8791	<0.001	2.926	11.901
Early-stage AD	Control	-4.4588*	1.6784	0.026	-8.467	-0.451
	Moderate-stage AD	2.9551	1.8548	0.254	-1.474	7.384
Moderate-stage AD	Control	-7.4138*	1.8791	<0.001	-11.901	-2.926
	Early-stage AD	-2.9551	1.8548	0.254	-7.384	1.474

* p<0.05;

95%CI: 95% confidence interval.

The mean CFF values of the participants with and without AD were compared according to gender, age group (<70 and ≥70), education level, and the number of drugs used, and there was no statistically significant difference (p>0.05) (Table 4).

**Table 4:** Comparison of mean Critical Flicker Fusion value according to gender, age group, education level, and the number of drugs used by Alzheimer's disease and control groups.

			AD	Control
Mean CFF	Gender	Male	37.75±8.50 (n=26)	43.94±3.84 (n=13)
		Female	35.38±5.41 (n=32)	44.57±3.94 (n=12)
		p-value	0.203	0.686
	Age	<70	36.56±6.07 (n=29)	43.23±3.50 (n=16)
		≥70	36.34±7.94 (n=29)	46.06±3.88 (n=9)
		p-value	0.907	0.075
	Number of drug usage	<5	36.96±7.09 (n=26)	44.41±3.98 (n=14)
		≥5	35.80±7.90 (n=25)	43.77±4.18 (n=8)
		p-value	0.584	0.729
	Education	No formal education	35.57±6.67 (n=23)	44.82±3.66 (n=6)
		Primary school	37.62±6.71 (n=25)	43.81±3.69 (n=10)
		Secondary school and above	35.55±8.67 (n=10)	44.34±4.43 (n=9)
		p-value	0.550	0.883

AD: Alzheimer's disease; CFF: Critical Flicker Fusion.

**Figure 1:**
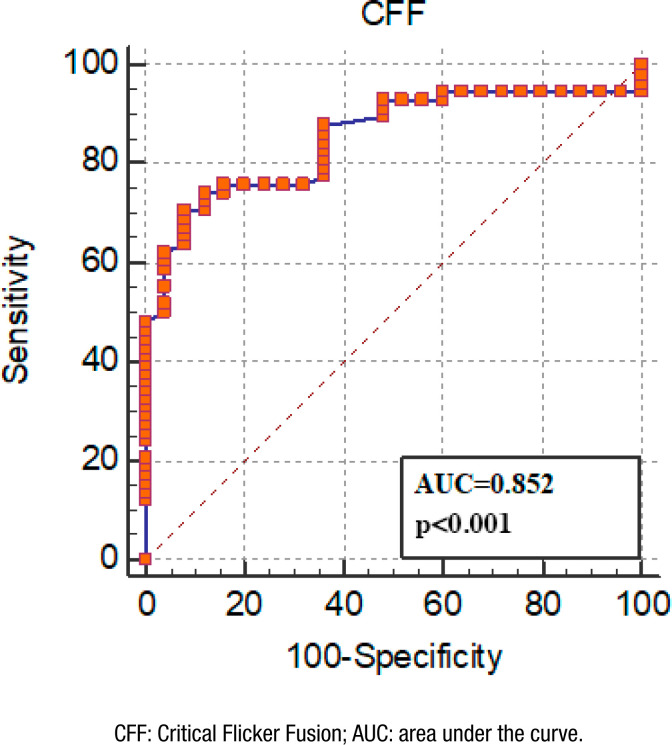
Receiver Operating Characteristic analysis.

The ROC analysis was used to determine the cutoff point for the CFF variable, such as 39 Hz [p<0.001; AUC=0.852; sensitivity=70.69% (95% confidence interval [95%CI] 57.3–81.9); specificity=92.00% (95%CI 74.00–99.00)] (Figure 1).

## DISCUSSION

From this study, by using neuropsychological methods, the current research was able to shed light on the characteristics of different stages of AD, and important findings and results were obtained to add to this field. First, we found that it is sensitive not only to distinguish AD cases from healthy controls but also to describe the progression of cognitive impairment at different stages of AD. Second, the neuropsychological protocol used was also sensitive to determine the cognitive characteristics at different stages of the AD spectrum. In addition, neurophysiological tests were correlated with the tests used to determine the diagnosis and stage of the disease.

CFF was used extensively to investigate the effects of psychoactive drugs on healthy volunteers about 50 years ago and has recently been shown to be important in detecting minimal hepatic encephalopathy in patients with liver cirrhosis^21^. The fact that the test is not affected by educational factors, cost-effective, and easy to manage at the same time makes it an “ideal” assessment tool. It can also be easily implemented by relatively poorly trained staff. Consequently, it is currently one of the most popular techniques in psychometric research.

CFF can be measured in two different ways, namely, ascending and descending thresholds. At the ascending threshold, the frequency of light is increased until flickering stops. At the descending threshold, the frequency of light is reduced from a high level until the onset of flickering appears. Consequently, the ascending and descending thresholds are averaged. CFF scores are normally distributed in healthy elderly individuals and do not change significantly in the age range of 60–90. However, due to increasing age and worsening central nervous system pathology (e.g., AD), its sensitivity changes in cognitive function, and it will be very difficult to interpret in the early stage of the disease^10^.

In a study by Curran et al., 26 AD patients were included, and CFFT and decreasing threshold were found to be significantly lower in AD patients compared with controls^8^. In another study, 46 patients were evaluated, and the only decreasing component of CFFT and verbal fluency test was significantly different in the two groups (AD vs. control group)^10^. A low descending threshold value of CFFT may be a characteristic feature of AD. Therefore, in our study, we calculated the descending threshold values of CFFT in the AD and control groups. The mean CFF values in the AD group were significantly lower than the controls. Individuals in both groups were homogeneously distributed according to age, gender, the number of drugs used, education level, and basic life habits. And there was no relationship between these factors and the CFF score. The standardized mini-mental test score of the AD group was significantly lower than the control group. The MMSE score was positively correlated with the mean CFFT value. When patients with AD were divided into two groups, such as early and moderate stages, and evaluated with the control group, there was a significant difference between the control group and the mean CFF values of the early-stage AD group and the moderate-stage AD group. As the disease stage increased, there was a decrease in CFFT score.

Current international guidelines have yet to confirm the use of electroencephalographic (EEG)/magnetoencephalographic (MEG) biomarkers in clinical studies in patients with AD, despite the increase in recently proven evidence. However, in several previous studies, a slowdown in brain activity associated with cognitive impairment, predicting the detection of dementia in the early stages, has been demonstrated using some neuropsychiatric tests^22-25^. Previous reports frequently used the tests, such as MEG and EEG, to investigate the neurophysiological features in AD and mild cognitive impairment (MCI) and gave the results consistent with our presented findings. In the seminal MEG study comparing AD and MCI patients and the control group, it was found that increased delta activity in the posterior parietal and precuneus cortices showed a negative correlation with the cognitive state^26^. Electrophysiology is a multiscale methodology suited to probing the effects of AD neuropathological processes and disease-modifying drugs on synchronization/desynchronization and coupling/decoupling of brain neural activity in preclinical and clinical research^27^. The lack of studies using neurophysiological markers in AD is remarkable, and the majority of published studies have focused on comparing groups of young or middle-aged patients with Down syndrome.

The CFF test is currently one of the most popular techniques in psychopharmacological research. Our study shows that CFF scores are normally distributed in healthy elderly individuals and do not change significantly with increasing age. Other benefits of the test are that it requires minimal training for testing and is relatively fast and easy to manage. It can also be used to monitor patients at high risk of developing AD more regularly.

As a result, we found a significant difference between CFF values of AD and healthy group in our study. The CFF test can play an important role in the early diagnosis of AD. Our study is the first and only study in the literature to give cutoff value for CFF in AD. The important features of the test are that its applicability is simple and practical, it is not invasive, it is cost-effective, and it does not differ according to age, gender, and education level. Considering the history and clinical findings, the CFF can be used as an important diagnostic tool in patients with AD. Also combined with clinical and other information, it can help patients report their decision to start an antidementia drug at an early or possibly preclinical stage to maximize any possible clinical benefit.

Future studies can be done with more cases. A study can be done compared with cases in the preclinical stage (amyloid detected in positron emission tomography [PET] imaging) and patients in other stages. In addition, the relationship between CFF scores and amyloid plaques on PET imaging and tau bodies in plasma or cerebrospinal fluid can be investigated.
